# A Heterozygous Genotype-Dependent Branched-Spike Wheat and the Potential Genetic Mechanism Revealed by Transcriptome Sequencing

**DOI:** 10.3390/biology10050437

**Published:** 2021-05-14

**Authors:** Tian Ma, Lei Li, Yang Zhao, Chen Hua, Zhengxi Sun, Tao Li

**Affiliations:** Key Laboratory of Plant Functional Genomics of the Ministry of Education, Jiangsu Key Laboratory of Crop Genomics and Molecular Breeding, Collaborative Innovation of Modern Crops and Food Crops in Jiangsu, Jiangsu Key Laboratory of Crop Genetics and Physiology, College of Agriculture, Yangzhou University, Yangzhou 225009, China; tianma598@gmail.com (T.M.); lilei@yzu.edu.cn (L.L.); zy18252737418@gmail.com (Y.Z.); chenhua0729@foxmail.com (C.H.); zhengxisun@yzu.edu.cn (Z.S.)

**Keywords:** wheat, branched spikelets, genetic pattern, gametophytic male sterility, transcriptome sequencing

## Abstract

**Simple Summary:**

This paper reported a novel type of branched spike wheat from a natural mutation event. The branched spike was controlled by a heterozygous genotype. The genetic patterns showed that gametophytic male sterility probably contributed to the heterozygous genotype responsible for the branched spike trait. Expressional levels and *Wheat FRIZZY PANICLE* (*WFZP*) sequencing between the mutant with the branched spike and the wild-type with the normal spike showed that *WFZP* was not the causal gene for the branched spike. Data from transcriptome sequencing indicated that carbohydrate metabolism might be involved in the formation of the branched spike trait.

**Abstract:**

Wheat (*Triticum aestivum* L.) spike architecture is an important trait associated with spike development and grain yield. Here, we report a naturally occurring wheat mutant with branched spikelets (BSL) from its wild-type YD-16, which has a normal spike trait and confers a moderate level of resistance to wheat Fusarium head blight (FHB). The lateral meristems positioned at the basal parts of the rachis node of the BSL mutant develop into ramified spikelets characterized as multiple spikelets. The BSL mutant shows three to four-day longer growth period but less 1000-grain weight than the wild type, and it becomes highly susceptible to FHB infection, indicating that the locus controlling the BSL trait may have undergone an intensively artificial and/or natural selection in modern breeding process. The self-pollinated descendants of the lines with the BSL trait consistently segregated with an equal ratio of branched and normal spikelets (NSL) wheat, and homozygotes with the BSL trait could not be achieved even after nine cycles of self-pollination. Distinct segregation patterns both from the self-pollinated progenies of the BSL plants and from the reciprocal crosses between the BSL plants with their sister NSL plants suggested that gametophytic male sterility was probably associated with the heterozygosity for the BSL trait. Transcriptome sequencing of the RNA bulks contrasting in the two types of spike trait at the heading stage indicated that the genes on chromosome 2D_S_ may be critical for the BSL trait formation since 329 out of 2540 differentially expressed genes (DEGs) were located on that chromosome, and most of them were down-regulated. Kyoto Encyclopedia of Genes and Genomes (KEGG) analysis showed that carbohydrate metabolism may be involved in the BSL trait expression. This work provides valuable clues into understanding development and domestication of wheat spike as well as the association of the BSL trait with FHB susceptibility.

## 1. Introduction

Wheat (*Triticum aestivum* L.) is one of the world’s most widely cultivated crops, and it feeds nearly 30% of the world population [1]. The spike architecture of wheat, also known as the compound inflorescence, is normally composed of sessile spikelets arranged in two opposite rows along the main axis (the rachis), with each spikelet producing three to five florets [2]. Branched wheat has additional spikelets on an extended rachilla that develop from the axillary region, which have more spikelets and kernels per spike. In cereal crops, grain yield depends mainly on number of spikes per unit crop area, number of grains per spike, and grain weight. Of these factors, grain number per spike is influenced by inflorescence architecture and floral development processes [3].

Plant development depends on the activity of various types of meristems that generate organs such as leaves and floral organs throughout the life cycle. All shoot parts of the plant, such as leaves, stems, and flowers, develop from the shoot apical meristem. Therefore, the regulation of meristem maintenance and fate is very important for plant growth [4]. Endogenous hormones regulate young wheat spike development and affect the number of spikelets by regulating the duration of unilateral and dimorphic differentiation of wheat [5], and they regulate the development of floret [6]. The morphological diversity of inflorescences suggests that different genes or homologous genes with divergent functions may be involved in the spike development of different crop species. Wheat floral organs provide the basis for grain formation such that wheat yield and quality are directly influenced by floral organ development [7].

The branched spike in wheat was discovered nearly as early as one century ago [8]. The reproductive spikelet develops as a terminal meristem on the spike and later differentiates into the floral meristem. If the floral meristem develops as a lateral spikelet instead of a floret, a branched spikelet is produced [9]. Genetic studies have demonstrated that the *TtBH/ Wheat FRIZZY PANICLE* (*WFZP*) gene determines the formation of the lateral meristem in wheat, and this gene is orthologous to the *AP2/EREBP*-like transcription factor genes responsible for branched silkless1 (*BD1*) in maize and for frizzy panicle (*FZP*) in rice [10,11]. Several key regulators in spike development in rice and maize have been identified [12,13]; however, the underlying regulatory mechanisms remain to be fully characterized. Very few advances have been made toward understanding spike development in wheat probably due to its polyploidy, large genome size (17 Gb), and low transformation efficiency. Supernumerary spikelets were reported to be controlled by two recessive genes [14], which were mapped on 2A and 2B chromosomes, and a strong suppressor gene for branched spike traits was identified on 2D chromosome [15]. The mutants with supernumerary spikelets or branched spike are valuable for studies on wheat spike development, yield trait, wheat domestication, and the association with wheat disease on head (for example, FHB). However, the key regulators and genetic networks involved in wheat spike development are still largely unknown.

In this paper, we reported a heterozygous wheat mutant with branched spikelet (BSL) trait, which occurred from wheat line YD-16 with a normal spike and moderate resistance to FHB. The objectives of this study were to explore the genetic pattern, agronomic performance, FHB resistance, and potential molecular basis underlying the BSL trait through RNA sequencing.

## 2. Materials and Methods

### 2.1. Evaluation of Agronomic Traits and FHB Resistance of the BSL

**Figure 1:**
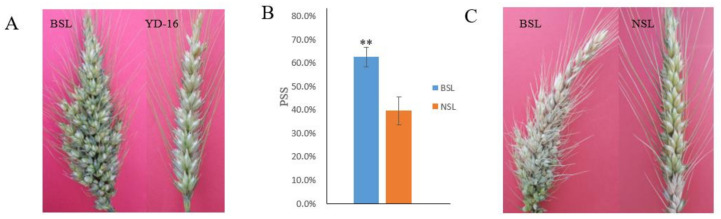
Comparisons of the spike architecture and FHB resistance of the BSL and the NSL plants. The mutant has branched spikelets (BSL), and the wild type has normal spikelets (NSL). (**A**) Spike morphological characteristics of the BSL mutant and the wild-type YD-16. (**B**,**C**) Contrasting FHB resistance levels of the BSL plants and the NSL plants after artificial inoculation. The PSS was calculated by infected spikelets/total spikelets in 21 days after inoculation.

YD-16 is a wheat breeding line developed in Yangzhou University, China, and it has a normal spike trait and confers a moderate level of resistance to FHB. A naturally occurring mutation of the wheat line YD-16 generated a BSL mutant (M_0_) in 2010 (Figure 1A). The inbred progenies (M_1_) of the M_0_ mutant were segregated in spike architecture in the year of 2011. We randomly selected several BSL plants (M_1_) to develop inbred lines (M_2_) in 2012. The inbred descendants of the BSL plants were continuously segregated with normal spikelet (NSL) trait and BSL trait for consecutive five cycles of self-pollination (M_3_ to M_7_) and a homozygote for the BSL lines has not been identified ever. In order to achieve accurate segregation ratio of BSL plants to NSL plants from the inbred progenies from a BSL plant, we then randomly selected seven BSL plants (M_7_) to score segregation ratio for two consecutive years of 2018 (M_8_) and 2019 (M_9_). The panicles emerging from the sheath were bagged with paper bags prior to anthesis to prevent cross-pollination. A total of 1913 plants were produced from the seven BSL plants. Heading date, flowering date, number of grains per spike, and 1000-grain weight were compared between the BSL and NSL wheat plants. To verify if the changed spike architecture was accompanied by alteration for FHB resistance, the BSL and NSL plants were evaluated for FHB resistance by injecting about 100 conidiospores of *Fusarium graminearum* into a central spikelet of a spike during early anthesis and then were bagged with a plastic sandwich bag to keep humidity. The plastic bag was removed in 72 h after inoculation. The proportion of symptomatic spikeletes (PSS) was calculated by infected spikelets/total spikelets in 21 days after inoculation.

### 2.2. Genetic Pattern Analysis

A homozygous BSL line has not been achieved ever after nine cycles of self-pollination (M_1_ to M_9_). To investigate the genetic pattern of the BSL trait, we made reciprocal crosses between two sister individual plants contrasting in spike architecture in the selected inbred BSL lines. Pollen fertility was also examined by staining with KI solution and pictures were taken under a stereo microscope (MVX10, OLYMPUS Company, Japan).

### 2.3. Transcriptome Sequencing and DEGs Analysis

At the heading stage, the total RNA of the BSL and NSL plants from the seven M_9_ lines (derived from the seven independent M_7_ plants) were extracted from the bulked spikelets (three spikelets each line) contrasting in spike architecture, using the TRIzol reagent (Invitrogen, Waltham, MA USA). The total RNA was prepared with two biological replicates. DNA was removed using DNase (Invitrogen, Waltham, MA USA) and then cleaned using the RNAeasy Mini Kit (Qiagen, Germantown, MD, USA). The RIN (RNA integrity number) values (>8.0) of these samples were assessed using an Agilent 2100 Bioanalyzer (Santa Clara, CA, USA). The cDNA libraries for the BSL plants, and the NSL plants were constructed and sequenced by the Beijing Genomics Institution (Beijing, China). The sequenced data were assembled against the reference genome sequences of the bread wheat variety Chinese Spring (IWGSC RefSeq v1.0 assembly), and DEGs, KEGG, Gene Ontology (GO), and SNP (single nucleotide polymorphism) analysis were subsequently analyzed. HISAT (Hierarchical Indexing for Spliced Alignment of Transcripts) was used to do the mapping step and Bowtie2 [16] was used to map clean reads to the reference; then, the gene expression level was calculated with RSEM [17]. DEGs were detected with DEseq2 (Fold Change ≥ 2.00 and Adjusted *p* value ≤ 0.05) [18] and PossionDis (Fold Change ≥ 2.00 and FDR ≤ 0.001) [19].

GO annotation (http://geneontology.org/, accessed on 30 March 2021) and KEGG annotation (https://www.kegg.jp/, accessed on 30 March 2021) were applied to classify the DEGs. Getorf software (http://emboss.bioinformatics.nl/cgi-bin/emboss/getorf, accessed on 30 March 2021) was used to identify the open reading frame (ORF) of each unigene. Then, the ORFs were aligned to the transcription factor (TF) domains using hmmsearch software (http://hmmer.org/, accessed on 30 March 2021) [20]. The SNPs from the transcriptome sequencing of the BSL and NSL bulks were analyzed by software SOAPsnp [21]. The Blast program was used to compare the transcripts to the reference genome to obtain the SNPs information. A panel of 93 CAPS/dCAPS (derived/cleaved amplified polymorphic sequences) markers were designed in a high-throughput way using the software CAPS/dCAPS Designer [22]. The *Taq* enzyme was used for polymerase chain reaction (PCR) and the amplified products were digested by the corresponding restriction enzymes. After enzyme digestion, electrophoresis was performed in agarose gel. The labeling results were divided into four categories: ‘M’, ‘F’, ‘×’, and ‘√’. ‘M’ means that two or more bands of different sizes were produced after digestion with corresponding restricted enzyme; ‘F’ means failure of the PCR amplification; ‘×’ and ‘√’ are monomorphic and polymorphic between the BSL and NSL wheat plants, respectively [23].

### 2.4. Quantitative Real-Time PCR (qRT-PCR) Analysis

At heading stage, total RNA was extracted from spikelets of the BSL and NSL wheat plants. The RNA integrity and purity were determined by agarose gel electrophoresis and One-Drop spectrophotometer (A260/280 > 1.9, A260/230 > 2.0), respectively. Then, total RNA (1 ug) was treated with RNase free DNase, and the first-strand cDNA was synthesized by reverse transcription using an oligo (dT) primer (Takara, Japan). In the qRT-PCR experiments, the wheat Actin gene and 20 DEGs randomly selected were amplified with corresponding gene-specific primers (Table S1). Amplification reactions were prepared using the SYBR Prime Script RT-PCR Kit (Takara, Japan). The threshold cycle (Ct) was determined by using the maximum-second derivative function of the software for the ABI 7900HT Real-Time PCR System (Applied Biosystems, Foster, CA, USA). Three biological replicates were prepared for each type of spike trait. Experimental errors were calculated from the standard deviation among the three biological replicates. Verification of the key DEGs with differences of fold change ≥2 in representative pathways was performed as described above, and the list of specific primer sequences is in Table S1.

## 3. Results

### 3.1. The BSL Plant Is a Naturally Occurring Mutant 

YD-16 is a wheat breeding line with a normal spike trait and confers a moderate level of resistance to FHB. We found a BSL mutant from a wheat inbred line YD-16 (Figure 1A). In order to clarify if the BSL mutant is derived from natural mutation rather than from outcrossing, molecular markers were used to check the genetic similarities of the BSL and the wild type with NSL trait. The transcripts sequence (about 200 Mb with 100) of BSL and NSL bulks were compared to the wheat reference genome, and 7175 SNPs on 21 chromosomes between the BSL and NSL bulks were identified. We considered that wheat is a polyploid species with a complex genome, and the false positive SNPs frequently occurred. Ninety-three SNPs in the whole genome were randomly selected and then transformed into CAPS/dCAPS markers by CAPS/dCAPS designer. The results showed that 34 out of 93 markers failed to produce any bands, 15 markers produced two or more fragments of unexpected sizes (Table S2, Figure S1), and 44 markers produced clear target bands, but they were monomorphic between the BSL and NSL plants, suggesting that most of the SNPs between the BSL and NSL bulks were false positive and the BSL trait was from a natural mutation rather than outcrossing.

### 3.2. The Genetic Patterns of the BSL Trait

The inbred offspring from the plants with BSL trait continuously segregated into BSL plants and NSL plants, whereas the offspring of the NSL wheat plants derived from the inbred BSL plants had normal spike traits. Segregation ratios of BSL plants to NSL plants derived from the inbred lines from randomly selected seven BSL plants in years of 2018 and 2019 were counted. In 2018, a total of 989 plants from the seven BSL plants (M_7_) were produced, with 504 for BSL and 485 for NSL plants, according a segregation ratio of 1:1 (χ^2^ = 0.328).

In 2019, we selected one BSL plant from each of the seven inbred lines (M_8_) to advance the BSL inbred lines to a higher generation (M_9_), and for comparison, one NSL wheat plant was also randomly selected from each population of inbred lines developed from an NSL wheat plant (M_8_) to investigate the segregation of spike traits. All the inbred progenies from the NSL plant showed normal spike type (750 NSL: 0 BSL), while the 924 inbred progenies from the seven BSL plants continued to segregate, with 474 for BSL plant and 450 for NSL plants, which is consistent with ratio of 1:1 (χ^2^ = 0.573).

A total of 1913 plants were generated from the seven populations of inbred lines from the BSL plants in two consecutive years (2018 and 2019). The numbers of branched and normal spike plants were segregated with a ratio of 1:1 (Table 1). The consistent segregation ratio of 1:1 of the descendants in spike morphology indicated that the BSL trait was controlled by a heterozygous genotype. Reciprocal crosses between a BSL plant with an NSL sister plant were then made to understand the segregation patterns in the spike traits. The F_1_ progenies with the branched and the normal spike were segregated by a 1:1 ratio (20 NSL: 23 BSL, χ^2^ = 0.0930 < 3.84) when the BSL wheat plant served as a maternal parent; otherwise, all the offspring showed normal spike traits when the BSL plant served as the paternal parent. Pollen fertility examination showed that there was no difference in pollen fertility between the BSL and the NSL plants (Figure S2).

The Chi-square test indicated the segregation ratio of BSL and NSL plants accorded with 1:1 (χ^2^_0.05_ = 3.84).

### 3.3. The BSL Mutant Significantly Differed from the NSL Plants in Agronomic Traits and FHB Resistance 

Significant differences in FHB resistance, heading and flowering dates, number of grains per spike, and 1000-grain weight were observed between the BSL and NSL plants (Table 2). The average days from sowing to heading of the BSL plants were three to four days longer than the NSL plants, and average days from sowing to anthesis of the BSL plants were four days longer than the NSL plants (Table 2). The BSL plants had more grains per spike but significantly lower 1000-grain weight than the NSL plants. The mean FHB severity PSS of the BSL plants (62.5% ± 4.2%) was significantly (*p* < 0.01) higher than that of the NSL plants (39.5% ± 6.0%) (Figure 1B,C).

### 3.4. DEGs between the BSL Plants and the NSL Plants

**Figure 2:**
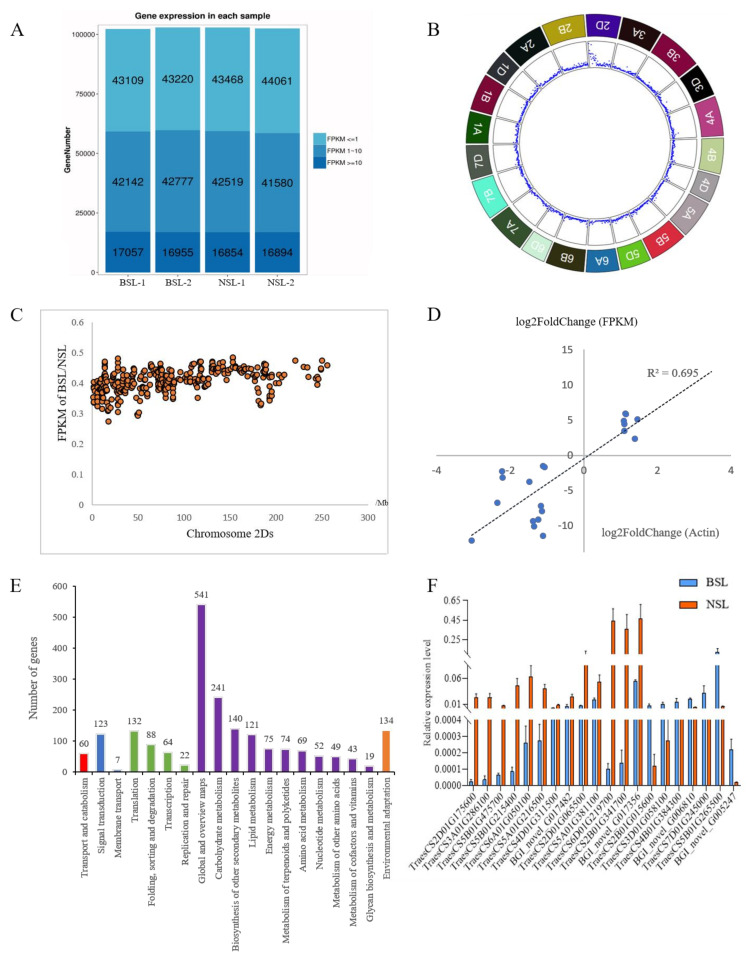
Analysis of DEGs expressions. (**A**) Levels of the expressions of all the genes detected in transcriptome sequencing of the BSL and the NSL bulks. (**B**) The density of DEGs on wheat chromosomes. (**C**) DEGs on 2Ds chromosome. Each dot represents the ratio of FPKM of a gene in the BSL plants when compared to the NSL plants. (**D**) Significant correlations between RNA-seq and qRT-PCR with Actin as a reference. (**E**) KEGG analysis of DEGs in the BSL and the NSL bulks. (**F**) Verification of 20 key transcripts in the representative pathways by qRT-PCR.

RNA sequencing was adopted to understand the genes involved in the BSL trait formation. After trimming the adaptor sequences, ambiguous reads, and low-quality reads, a total number of 220.5 million paired-end reads accounting for 22.05 Gb of the clean data were generated for the BSL and the NSL libraries. The Q30 percentage (the percentage of bases in the reads with a phred quality equal or bigger than 30) of the clean reads were higher than 90%, suggesting that the sequencing results were valid (Table S3). The expression levels of the majority of genes were low (Fragments Per Kilobase Million (FPKM) ≤ 1) to moderate (with FPKM between 1 and 10) (Figure 2A). Through transcriptome sequencing, 106,377 and 106,467 genes were detected in the BSL and the NSL bulks, respectively, among which 96% (102,867) genes were common across the two bulks, indicating that the coverage of transcripts was very good.

A total of 2540 DEGs were found between the BSL and the NSL bulks (Table S4, Figure S3), and 329 DEGs were located on chromosome 2D_S_ (Figure 2B,C). The average number of DEGs was 1.22/Mb on 2D_S_, while the density of DEGs on the other chromosomes was 0.15/Mb. Further analysis showed that 328 DEGs from the BSL bulk were down-regulated on chromosome 2D_S_ and the average FPKM of the BSL/the NSL was 0.41.

Since the mutated gene had pleiotropic effects on the several traits mentioned above, transcription factors (TFs) might be associated with the expression of the BSL trait. In this study, a total of 161 TFs were found to be associated with plant development. These TFs belong to 25 TF families such as AP2, bHLH, MYB, bZIP, GRAS, and WRKY (Table S5). The majority (116) of the TFs were down-regulated. Four AP2 TFs significantly down-regulated were annotated to ethylene-responsive transcription factor, which were located on 1A, 1B, 1D, and 2B chromosomes. The other TFs detected in the AP2 TF family showed no expressional difference between the BSL and the NSL bulks. To validate the gene expression, qRT-PCR was performed for 20 randomly selected transcripts with differential expression levels between the BSL and the NSL plants at the heading stage (with the actin gene as a reference). The oligonucleotide primer sequences used to quantify the transcripts for individual genes are listed in Table S1. The FPKM of RNA-seq and relative expression level with actin as a reference of qRT-PCR were highly correlated (*R*^2^ = 0.695) (Figure 2D).

### 3.5. Carbohydrate Metabolism Showed a Significant Difference between the BSL and the NSL Plants

To further explore the biological functions and interactions of the DEGs, KEGG pathway and transcript annotations were analyzed (Figure 2E). A total of 1077 DEGs were assigned to 116 KEGG pathways (Table S6). DEGs were significantly enriched in starch and sucrose metabolism (85), pentose and glucuronate interconversions (48), carbon fixation in photosynthetic organisms (30), sesquiterpenoid and triterpenoid biosynthesis (21), and the biosynthesis of secondary metabolites (296), respectively (Table S6). The majority of DEGs in the top five pathways mentioned above were down-regulated. To identify the functional category of the annotated transcripts, GO analysis was employed to classify the transcripts annotated by known proteins. A total of 1384 DEGs with significant similarity to known Uniprot proteins were assigned to GO terms; 1006 DEGs were assigned to the Molecular Function, followed by Cellular Component (996) and Biological Process (885). The hydrolase activity, acting on glycosyl bonds term in the Molecular Function, was significantly enriched (*p* < 0.05) (Table S7). In the Cellular Component, the external encapsulating structure, cell periphery, site of polarized growth, growing cell tip, and cell wall were significantly enriched (*p* < 0.05) (Table S8). In the Biological Process, a series of changes occur in the BSL plants and the response of cell development and differentiation terms such as cellular component morphogenesis, cell morphogenesis involved in differentiation, cellular developmental process, and pollen wall assembly were significantly enriched (*p* < 0.05) (Table S9).

We selected 20 key DEGs (Table S10) in the top five pathways with significant differences between the BSL and the NSL bulks for qRT-PCR validation. The results showed that four DEGs in the starch and sucrose metabolism pathway, four DEGs in the pentose and gluconate conversions pathway, three DEGs in the biosynthesis of secondary metals pathway, one DEG in the squiterpenoid and triterpenoid biosynthesis pathway, and one DEG in carbon fixation in the photosynthetic organizations pathway were significantly down-regulated in the BSL plants (Figure 2F) when compared with the NSL plants.

## 4. Discussion

### 4.1. The Gene(s) Responsible for the BSL Trait May Have Undergone Negatively Artificial Selection 

The BSL mutant had more grains per spike, smaller grain size, lower 1000-grain weight, about a three to four-day longer growth period, and higher FHB severity than the wild-type YD-16, which has a normal spike. These observations indicated that the gene(s) responsible for the BSL trait had pleiotropic effects on spike architecture, heading date, flowering date, yield trait, and FHB resistance. Considering that the commercial wheat cultivars worldwide have normal spikes, the gene (s) underlying the BSL trait may have undergone an artificially and (or) naturally negative selection, and the corresponding allelic variation controlling the normal spike should be an economically important hotspot for artificial domestication.

It was interesting that the wild-type YD-16 showed moderate resistance to FHB, whereas the BSL plants became highly susceptible, indicating that the spike architecture change was also associated with FHB resistance (this does not mean that all wheat genotypes with normal spike always associate with FHB resistance). Until now, only two QTL, *Fhb1* [24,25,26] and *Fhb7* [27], for FHB resistance were claimed to have been cloned, and neither of them were reported to be associated with spike architecture. Therefore, YD-16 most likely carries a novel type of FHB resistance gene. Mapping and cloning the gene for the BSL trait may unveil a novel type of mechanism for FHB resistance.

### 4.2. Gametophytic Male Sterility Putatively Contributed to the Heterozygosity of the BSL Trait

**Figure 3:**
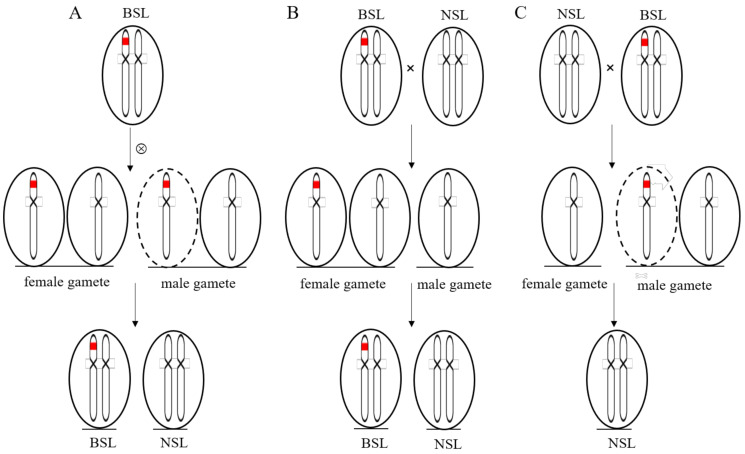
The genetic model of putative heterozygosity at the causal locus for the BSL trait. (**A**) The female gametes develop normally and the male gametes carrying the mutated gene for the branched spikelets aborted, resulting in a 1:1 segregating ratio of the BSL plants and the NSL plants in the offspring of selfed BSL plants. (**B**) The F_1_ progenies with the branched and the normal spike segregated by a 1:1 ratio when the BSL plants served as a maternal parent, and (**C**), all the offspring showed normal spike trait when the BSL plant served as paternal parent. The red bar represents the gene for the branched spikelets. The dotted ellipse represents aborted male gamete for the BSL plants.

Flowering plants have developed specialized structures for the production of male and female gametes. Development of anthers is a complex and precise biological process from the generation of sporogenous cells to the development of microspore mother cells, to meiosis, to microspore formation, and to maturation and pollination [28]. The abnormality of the development of anthers is the main reason causing male sterility in plant. Pollen fertility in wheat was reported to be associated with the ability to control and maintain sink strength and carbohydrate supply to anthers [29]. The offspring of the BSL plants always segregate into the NSL and the BSL with a ratio of 1:1, even after multiple generations of self-pollination, suggesting that the genetic locus responsible for the BSL trait should be heterozygous. Reciprocal crosses between the BSL and the NSL plants showed different segregation patterns of the offspring at F_1_ generation, with a ratio of 1:1 when the BSL plants served as a female parent, resembling the self-pollination consequences of the BSL plants; otherwise, all offspring had a normal spike when the BSL served as a male parent. These results suggesting the failure of male gametes of the BSL plants most likely contributed to the causal heterozygosity for the BSL trait (Figure 3). However, there was no significant difference in pollen fertility between the BSL and the NSL plants, indicating that gametophytic male sterility might occur at an earlier stage of pollen development, putatively between the first division of meiosis and mononuclear pollen formation. Therefore, the BSL mutant could be a novel type of branched wheat that has not been reported before.

### 4.3. Wfzp Was Not the Causal Gene for the BSL Mutant

Although various spike types are observed, such as a compact spike in *T. compactum* (club wheat) or a lax spike in spelt wheats (*T. aestivum* ssp. *spelta*), branching spike is rarely detected in hexaploid wheat [9]. It was reported that the supernumerary spikelet in wheat is controlled by one or two recessive genes [15,30,31,32]. It was also reported that the four-rowed spike and ramified spike were associated with a major recessive gene on chromosome 2A and numerous minor genes, including one on chromosome 2B [33,34]. The ‘triple-spikelet’ trait in a Tibetan landrace of bread wheat is dominantly determined by a major gene on chromosome 2A [35]. However, no gene for spike architecture in wheat has been cloned, except the wheat Q gene, which is related to spike shape, the variation in the free-threshing character and many other domestication-related traits such as glume toughness, rachis fragility, and spike length [36]. Previous reports showed a strong suppressor gene or an inhibitor for branched spike trait of common wheat was identified on 2D chromosome [15,34,37,38]. Interestingly, in the BSL plants, the chromosome 2D_S_ had the most DEGs down-regulated. Therefore, we hypothesized that down-regulated genes on 2D_S_ chromosome might be associated with branched spike development.

Spikelet meristems (SM) maintenance and the transition from SM to floral meristem (FM) are regulated by *FZP* in rice and its homologous gene *BRANCHED SILKLESS1* (*BD1*) in maize [39,40]. It was reported that *FZP* encodes an AP2/ERF transcription factor with transcriptional activator activity [39] and specifically expressed at the axils of rudimentary glumes primordial to control floral fate. In bread wheat, mutations in *WFZP* result in supernumerary spikelets phenotype showing multirow spike developed at a rachis node [41], and *WFZP* also functions as a transcriptional activator in wheat [42]. The *WFZP* gene determines the formation of the lateral meristem in wheat [10,11], which has two copies in wheat, one on 2A chromosome (*TraesCS2A02G116900*) and the other on 2D chromosome (*TraesCS2D02G118200*). However, only the copy on 2A chromosome (*TraesCS2A01G116900*) was detected in this study, and there was no significant difference in the expressional level between the BSL and the NSL plants. Further sequencing revealed that there was no sequence difference between the BSL and the NSL plants for *TraesCS2A01G116900* and *TraesCS2D02G118200*. Interestingly, we found four out of 622 AP2 TFs were annotated as ethylene responsive transcript, which were significantly down-regulated, and none of these four AP2 TFs were on chromosome 2A or 2D. So, we concluded that the BSL was most likely a new hexaploid branched wheat mutant, which may be controlled by a novel gene rather than *WFZP*.

### 4.4. Carbohydrate Metabolism Showed a Significant Difference between the BSL and the NSL Plants

Carbohydrates provide energy and nutrients for anthers development [43]. Abnormal carbohydrate metabolism (CM) may damage gametal development and lead to early abortion of male gamete [44] and the formation of branched spikes development. During grain development, sucrose is converted to starch, the major storage carbohydrate in wheat, which accounts for 80% of mature grain weight [45]. As part of CM, starch synthesis is of prime importance, since grain filling is mainly associated with this phenomenon [46], but other processes in which CM enzymes are involved are also important in several ways. However, most of the CM key enzymes were down-regulated in this study, including sucrose synthase (EC 2.4.1.13), hexokinase (EC:2.7.1.1), UTP-glucose-1-phosphate uridylyl transferase (EC:2.7.7.9), and endoglucanase (EC:3.2.1.4). DEGs were collected from the BSL and the NSL bulks at the heading stage before grain filling stage, so the down-regulation of CM key enzymes was not surprising, which corresponded with the plant growth stage. However, if the down-regulation of these enzymes occurred before the heading stage, it was likely to lead to early male gamete abortion; otherwise, it may lead to uneven grain size and low 1000-grain weight if these enzymes continue to be down-regulated after the heading stage. Further research needs to be done to clarify these speculations in the future.

## 5. Conclusions

In this study, we reported a novel type of branched spike wheat that was probably controlled by a heterozygous genotype, and the BSL trait seemed to result from the within-allelic interaction between the mutated gene and the wild-type allele. Based on genetic patterns, we also concluded that gametophytic male sterility probably contributed to the heterozygous genotype responsible for the BSL trait. Contrasting agronomic performances between the BSL and the NSL plants suggested that the allelic variation from the wild type against the BSL trait might have undergone artificial positive selections during wheat domestication history. Expressional levels and *WFZP* sequencing between the BSL and NSL plants showed that *WFZP* was not the causal gene for the BSL trait in current study. Carbohydrate metabolism might be involved in the formation of the BSL traits. Mapping and cloning the gene underlying the BSL trait, which is in progress in our lab, would help decipher the relationships of spike development with wheat domestication and evolution as well as with FHB resistance.

## Figures and Tables

**Table 1:** Segregation ratio of BSL and NSL plants in the inbred progenies from BSL plants in the years of 2018 and 2019.

Year	Entry	Number of BSL Plants	Number of NSL Plants	χ^2^
2018	1	72	68	0.064
	2	74	75	0.000
	3	72	69	0.028
	4	77	76	0.000
	5	62	71	0.481
	6	73	53	2.865
	7	74	73	0.000
	Total	504	485	0.328
2019	1	74	67	0.255
	2	77	59	2.125
	3	78	77	0.000
	4	71	73	0.007
	5	51	55	0.085
	6	54	52	0.009
	7	69	67	0.007
	Total	474	450	0.573

**Table 2:** Agronomic traits of the BSL and the NSL plants.

	Days from Sowing to Heading	Days from Sowing to Anthesis	Grains Number per Spike	1000-Grain Weight/g
NSL	173.4 ± 1.35	177.2 ± 2.02	44.0 ± 12.98	47.5 ± 14.91
BSL	175.6 ± 1.56 **	180.5 ± 1.72 **	55.9 ± 14.70 **	35.6 ± 5.74 **

Data are expressed as mean ± SD. The statistical analysis of variance was determined by Dunnett’s test. ** is *p* < 0.01 when compared with NSL plants.

## Data Availability

The data presented in this study are available on request from the corresponding author.

## Supplementary Materials

The following are available online at https://www.mdpi.com/article/10.3390/biology10050437/s1, Figure S1: Validation of genetic background by CAPS/dCAPS markers of the BSL and the wild-type with NSL trait, Figure S2: Examination of the pollen fertility of the BSL and NSL plants, Figure S3: Volcano map of DEGs, Table S1: Primer sequences used to quantify the DEGs, Table S2: Validation of 93 CAPS/dCAPS markers between the BSL mutant and the wild-type, Table S3: Clean reads quality metrics, Table S4: 2540 DEGs between the BSL and NSL plants, Table S5: Differentially expressed transcripts between the BSL and NSL plants, Table S6: Significantly enriched KEGG pathways in the BSL plants, Table S7: Significantly enriched molecular function in the BSL plants, Table S8: Significantly enriched cellular components in the BSL plants, Table S9: Significantly enriched biological processes in the BSL plants, Table S10: Function annotation and expression of 20 key DEGs in five representative pathways.

## Abbreviations

BSL	Branched spikelets
NSL	Normal spikelets
FHB	Fusarium head blight
PSS	Proportion of symptomatic spikeletes
DEGs	Differentially expressed genes
WFZP	Wheat FRIZZY PANICLE
CAPS/dCAPS	Derived/cleaved amplified polymorphic sequences
CM	Carbohydrate metabolism
GO	Gene Ontology
