# Supplementary material for: A Heterozygous Genotype-Dependent Branched-Spike Wheat and the Potential Genetic Mechanism Revealed by Transcriptome Sequencing
Source: Biology (Basel). 2021 May 14;10(5):437. doi: 10.3390/biology10050437 (PMC8157103; doi:10.3390/biology10050437)
Supplement: Supplementary file 1 [file biology-10-00437-s001.zip › Supplementary figures and tables/Supplementary Figures..pptx]

## Slide 1
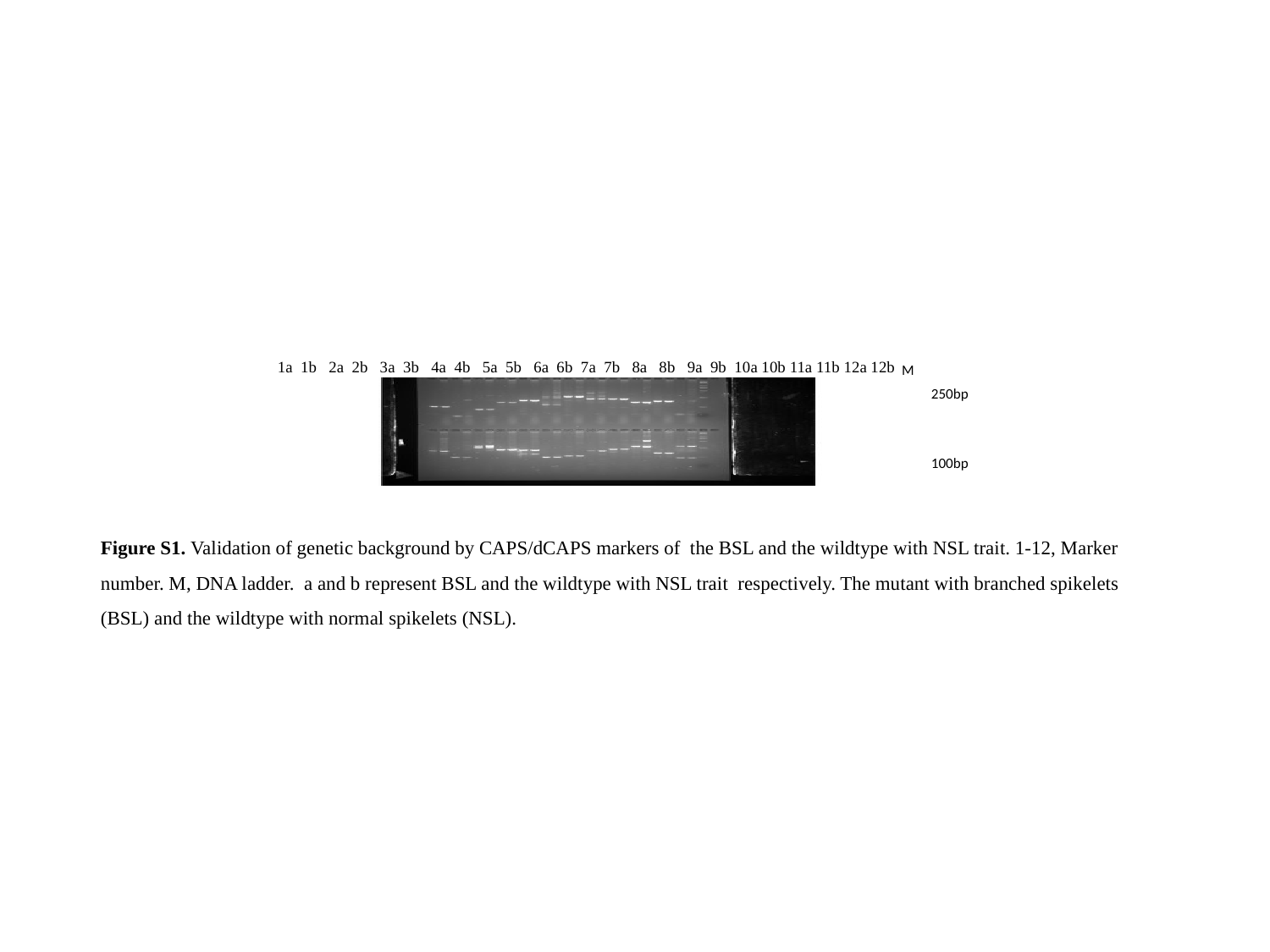

F8
M
250bp
100bp
 1a 1b 2a 2b 3a 3b 4a 4b 5a 5b 6a 6b 7a 7b 8a 8b 9a 9b 10a 10b 11a 11b 12a 12b
Figure S1. Validation of genetic background by CAPS/dCAPS markers of the BSL and the wildtype with NSL trait. 1-12, Marker number. M, DNA ladder. a and b represent BSL and the wildtype with NSL trait respectively. The mutant with branched spikelets (BSL) and the wildtype with normal spikelets (NSL).

## Slide 2
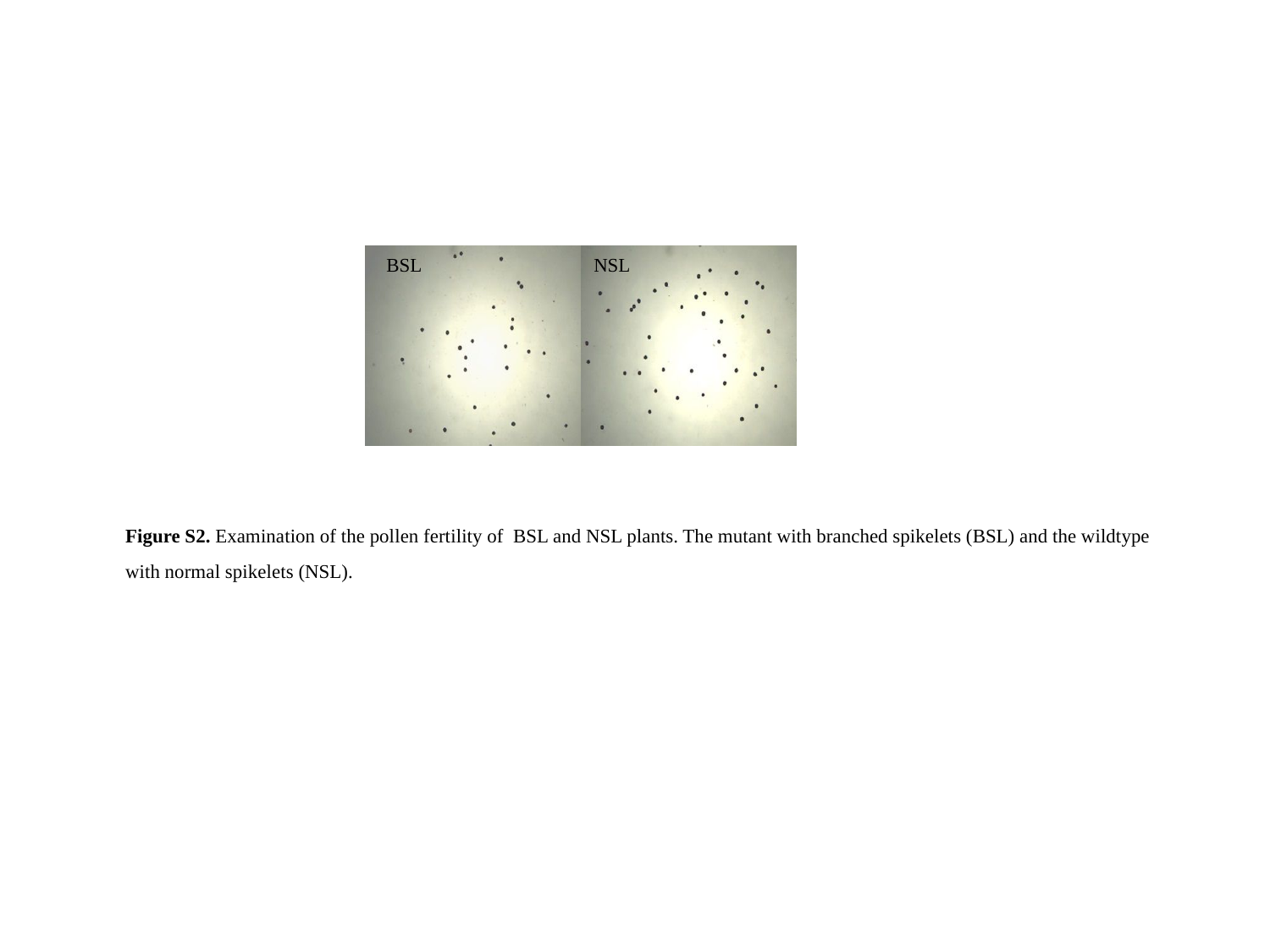

BSL
NSL
Figure S2. Examination of the pollen fertility of BSL and NSL plants. The mutant with branched spikelets (BSL) and the wildtype with normal spikelets (NSL).

## Slide 3
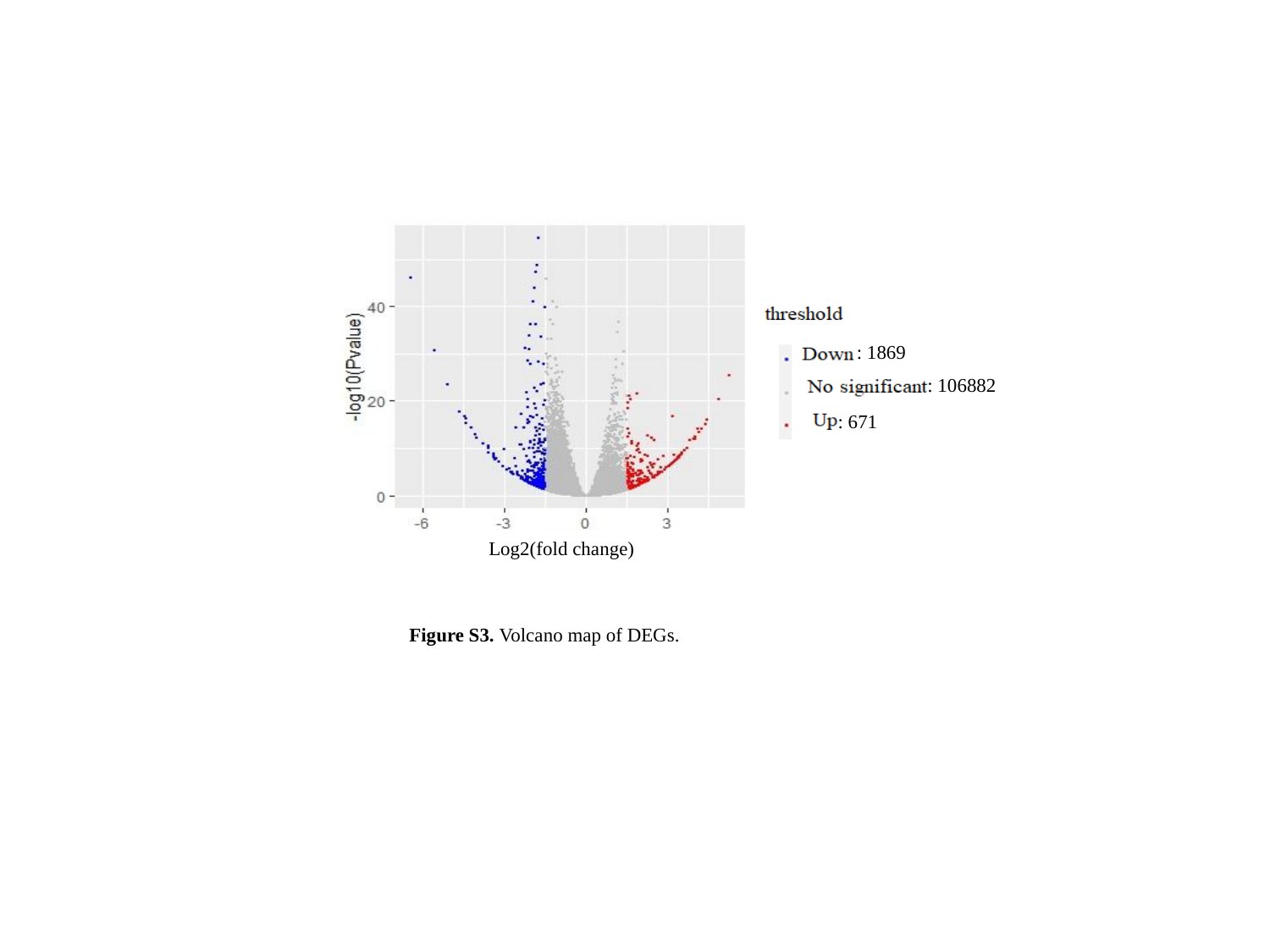

: 1869
: 106882
: 671
Log2(fold change)
Figure S3. Volcano map of DEGs.
